# Factor Structure and Psychometric Properties of the Farsi Versions of Empathy and Systemizing Quotient: Short Forms 

**Published:** 2018-10

**Authors:** Masih Shafiei, Meng Chuan Lai, Amir Hossein Memari, Fatemeh Sadat Mirfazeli, Sahar Zarei, Pouria Moshayedi, Ramin Kordi

**Affiliations:** 1 Sports Medicine Research Center, Neuroscience Institute, Tehran University of Medical Sciences, Tehran, Iran.; 2 Department of Linguistics, University of Tübingen, Tübingen, Germany.; 3 Child and Youth Mental Health Collaborative at the Centre for Addiction and Mental Health and The Hospital for Sick Children, and Department of Psychiatry, University of Toronto, Toronto, Canada.; 4 Autism Research Centre, Department of Psychiatry, University of Cambridge, Cambridge, United Kingdom.; 5 Department of Psychiatry, National Taiwan University Hospital and College of Medicine, Taipei, Taiwan.; 6 School of Medicine, Tehran University of Medical Sciences, Tehran, Iran.; 7 Department of Neurology, University of Pittsburgh Medical Center, Pittsburgh, USA.

**Keywords:** *Empathy Quotient*, *Factor Structure*, *Farsi*, *Psychometric Properties*, *Systemizing Quotient*

## Abstract

**Objective:** We aimed to examine the validity and reliability of the empathy quotient (EQ) and systemizing quotient (SQ) in a Farsi-speaking population.

**Method** **:** This study explores the factor structure and psychometric properties of the Farsi translations of the 22-item version of EQ and the 25-item version of SQ among 542 young university students.

**Results: **Applying a cross-validation approach, a 14-item two-factor model and a 15-item four-factor model for the Farsi translations of the short versions of EQ and SQ, respectively, were extracted from the exploratory dataset using exploratory factor analysis (EFA). Confirmatory factor analysis (CFA) on the validation dataset confirmed the factor structures identified by EFA. In addition, acceptable internal consistency and test-retest reliability were demonstrated for the Farsi translations of the 14-item two-factor EQ model and the 15-item four-factor SQ model.

**Conclusion: **The results suggested further evidence in favor of the multi-factorial constructs of the EQ and SQ and validity and reliability of the scales.

The empathizing–systemizing (E–S) theory introduces empathizing (E) and systemizing (S) as the 2 key cognitive processes explaining the psychology of sex differences (1). E is defined as the drive to identify others’ emotions and thoughts (also referred to as “cognitive empathy”) and to respond to them accordingly (also known as “emotional empathy”). S, on the other hand, allows one to recognize the inanimate world and construct and control rule-based systems. E–S theory predicts that women tend to show a preference for E over S and men show a preference for S over E (2-5). 

To quantify E and S preferences, 2 self-report questionnaires, the Empathy Quotient (EQ) and Systemizing Quotient (SQ), were developed by Baron-Cohen (2, 3). These questionnaires have shown high validity and reasonable internal consistency and reliability across cultures and languages (6, 7). A growing body of evidence has supported the E-S theory prediction on sex differences: women outscore men on the total and on the subscale scores of the EQ, while men outperform women on the total and on the subscale scores of the SQ (8-10). 

The current evidence on the matter of factor structure and association between EQ and SQ is controversial. Some studies have favored Baron-Cohen’s suggestion that the EQ and SQ measure unifactorial constructs (11-14). However, others have suggested that the EQ and SQ are, in fact, multifactorial constructs (10, 15). Additionally, the current literature shows constraints on the replicability of the suggested models for the EQ and SQ. The validity of these models has been left uninvestigated (e.g., 10, 11), unsupported (e.g., 8, 13), or marginally supported (e.g., 9) by independent studies. The association between E and S is marked by disagreements in theory, i.e., whether E/S factor space is single-axis or dual-axis (for more details see: 16) and on an empirical level. Empirically, previous studies investigating normative populations have reported a small to moderate correlation (17), in a positive or negative direction, between the scores of the 2 measures. 

The disagreements on the factorial construct of the EQ and SQ are suggested to partly stem from cultural differences among the studied samples (18) and the use of paper-and-pencil versus online versions of the questionnaires (10). Besides, we propose that decisions made in most of the previous studies regarding a few important methodological issues in the implementation of factor analysis might have played a role in the observed disagreements. First, the use of “Little Jiffy” procedure (i.e., applying principal components analysis (PCA) with varimax rotation and retaining components based on eigenvalues > 1.0) has been reprimanded repeatedly since such a combination of decisions has been found to be prone to producing invalid or distorted results (19). Surprisingly, 5 of 6 studies that investigated the factor structure of the EQ, used the “Little Jiffy” in whole or in part (12, 13 and 20). This also holds true for one (13) of the 2 studies (along with Samson & Huber, 2010) that explored the factor structure of the SQ. Second, treating ordinal data, such as EQ/SQ data, as continuous can lead to severe bias in parameter estimates, standard errors, or fit indices in structural modeling (21). Such a mistreatment can almost constantly be found throughout the literature on the structural modeling of the EQ/SQ. Taken together, it would come as no surprise if a correctly specified model did not fit data well and as a result a plausible model was rejected or a model was introduced that could not be replicated by subsequent studies (19).

To address the above concerns, following solutions were suggested. First, EFA should be used instead of PCA, as latent variables underlying the EQ or SQ are to investigate. Second, oblique rotation seems the prior rotation method as factors underlying the EQ/SQ are expected to correlate (22). Third, the number-of-factor problem can be approached using Structural Equation Methods that can provide goodness-of-fit measures to compare among a number of candidate models when performing EFA. Fourth, to treat ordinal data appropriately, factor analysis should be conducted on polychoric correlation matrices as opposed to Pearson correlation. These decisions direct us towards robust weighted least squares (WLS) estimation methods for the factor analysis. Currently, it is suggested that mean- and variance-adjusted WLS (WLSMV) can allow a model to converge even with a small sample size, yield less biased standard errors, and provide more accurate factor loading estimates (21). At last, a cross-validation approach can be employed to demonstrate whether or not a model obtained by EFA is replicable on an independent sample. 

Research goal 1. — To explore the factor structure of the Farsi version of the EQ-short and the SQ-short.

Research goal 2. — To investigate internal consistency and test-retest reliability of the Farsi versions of the EQ–short and the SQ–short.

Research goal 3. — To test the association between scores of the Farsi version of the EQ–short and SQ–short.Research goal 4. — To assess whether women outscore men on the Farsi version of the EQ–short and men outperform women on the Farsi version of the SQ–short.

## Materials and Methods

***Participants***

A total of 542 university students volunteered for the present study. Participants were enrolled from 4 different fields of study: science (physical sciences, mathematics, and engineering), humanities, health, and sports. Prior to enrolling, written informed consent was obtained from all participants. The study protocol was approved by the Ethical Committee of Tehran University of Medical Sciences.

***Material***

The EQ–Short (22 items) and the SQ–Short (25 items) developed by Wakabayashi et al. (13) were employed in this study. These short forms have shown strong correlations with their original versions; moreover, high reliability, and Cronbach’s alpha coefficients of .90 and .89 have been reported for the EQ–Short and SQ–Short, respectively, when examining 1761 students from Cambridge University, UK (13). 

Items of these questionnaires come in a 4-point rating scale with anchors: (1) totally agree, (2) agree, (3) disagree, and (4) totally disagree. Each item’s rating is transformed into either 0, 1, or 2 and then accumulated altogether to calculate scores for the EQ–Short and SQ–Short. A strong empathizing/systemizing response to each item is scored 2 points, a slightly empathizing/systemizing response receives the score of 1, and a response that is neither empathizing nor systemizing receives the score of 0. Thus, the total scores can range from 0 to 44 for the EQ–Short and 0 to 50 for the SQ–Short.

Based on the standardized protocols (23, 24), the EQ–Short and the SQ–Short were separately translated into Farsi. All items from each questionnaire were translated from English to Farsi by 2 independent Farsi-English bilinguals. Inconsistencies between the 2 translations were spotted, resolved through discussions, and one Farsi translation was made which was back-translated to English by 2 English-Farsi bilinguals. Inconsistencies in the English translations were identified and resolved through comparisons with the original English version of the questionnaires. At the end, an expert panel (the translators, a psychologist, a methodologist, and the authors) was convened so the observed discrepancies during the translation and backward translations were discussed and settled. A preliminary Farsi version of the scales was produced and piloted on 20 university students; and the final Farsi versions of EQ–Short and SQ-short, (EQ-Short–F and SQ-Short–F) were developed according to the findings of the pilot study.

***Analysis***

Factor analysis was conducted using Mplus 7 for windows. All other statistical analyses were performed by SPSS software Version 16 (SPSS, Inc., Chicago, Illinois). Applying a cross-validation approach, participants were randomly sorted into either an exploratory or a validation dataset with equal sizes (n = 271). EFA was conducted on the exploratory dataset to identify latent variables underlying the EQ-Short–F. CFA was performed on the validation dataset to assess the replicability of the constructs obtained by EFA. Test-retest reliability of the questionnaires was examined using a subgroup of 20 participants across the 2 split-half datasets who completed the questionnaires on 2 occasions (4–6 weeks apart).

Factor analysis was performed on the polychoric correlation matrix, generated between all possible pairs of items of each questionnaire. WLSMV was used as estimation method. Oblique rotation was applied when necessary. In the exploratory mode, data were fitted into a number of models differing in their number-of-factor. Number-of-factor ranged from 1 (i.e., a unifactorial model) to a maximum number, which was obtained according to the eigenvalue-greater-than-one rule and scree plot (21). Those models that had root mean square error of approximation (RMSEA) above the accepted threshold (0.05 or 0.06) were selected for further investigation (24). Modifications were performed on the selected models through which those items with unacceptable (< 0.35) or insignificant pattern/structure coefficient or cross-loading were allowed to be excluded from the questionnaire. Data on the remaining items was fitted onto the same model again and RMSEA was obtained. This process was continued until the model of best fit was identified. The model of best fit was the one having not only a simple and stable structure but also intuitive interpretability and reasonable validity and reliability when compared with competing models. Additionally, the number of indicators for each factor was kept equal or greater than the recommended value of 3 (21). To mark a model as the best fit when performing CFA, the following goodness-of-fit indices have to be met: a preferably non-significant chi-square test (χ2), a comparative fit index (CFI), and a Tucker-Lewis index (TLI) close to or greater than 0.95, an RMSEA below 0.05 or 0.06, and a weighted root mean square residual (WRMR) less than 1.0 (25, 26). 

## Results

Their mean age was 22.1 years (SD = 2.3; range = 17–28). Men (mean age, 22.4 years, SD = 2.3) and women constituted 37% and 63% of the sample, respectively.

**1** **.** **Factor Analysis**

*1.1* *.* *EQ-Short–F*

1.1.1. Exploratory Factor Analysis: Univariate score distributions from the exploratory dataset appeared to be moderately asymmetric since the largest skew was –1.46 and the largest kurtosis was 2.18, indicating assumption violation on multivariate normality.

Using eigenvalue and scree plot, EFA revealed that 1 or 2 factors should be retained. Therefore, models with a number of factors ranging from 1 to 3 were fitted into the data. The RMSEA yielded results of 0.08, 0.06, and 0.06 for one-, two-, and three-factor models, respectively, with no negative residual variances. According to RMSEA value (≤ 0.06), the two- and three-factor models were suggested to be appropriate. 

Regarding the two-factor promax-rotated model, factor 1 was composed of 3 social skills items (items 3, 4, and 12) and 3 emotional reactivity items (items 7, 11, 17). Thus, factor 1 was labeled as “social/emotional”. Factor 2 consisted of 8 cognitive empathy items (6, 9, 10, 16, 18, 19, 20 and 21), so it was regarded as “cognitive empathy”. The rest of items were excluded from subsequent analysis, as they did not load on either of the factors (items 2, 5, 22) or showed cross-loadings (items 1, 8, 13, 14, 15). RMSEA value for the remaining 14 items loaded onto 2 factors dropped from 0.06 to 0.05, representing a significant improvement in the model fit.

Regarding the three-factor promax-rotated model, 3 (items 1, 14, and 16) and 6 (items 6, 9, 18, 19, 20, 21) “cognitive empathy” items constituted factors 1 and 3, respectively. Factor 2 consisted of 3 “social skills” items (items 3, 4, 12) and 3 “emotional reactivity” items (items 7, 11, 17). The remaining items showed unacceptable pattern coefficients (items 2, 5, 22) or cross-loading (items 10 and 13), which were eliminated from the subsequent analyses. RMSEA value for the remaining 17 items loaded onto 3 factors remained equal to 0.06. Due to the new promax-rotated pattern coefficients, items 8 and 15 showed cross-loading and were removed from the analysis. RMSEA for three-factor model with the remaining 15 items decreased from 0.06 to 0.05, showing significant improvement in the fit. However, this model appeared to be problematic in factor interpretability, as it had 2 cognitive empathy factors. Hence, we rejected the model and selected the two-factor model with 14 items as the best fit model (Table 1). Factors 1 and 2 were significantly intercorrelated at r = 0.28 (Pearson Correlation; Table 1).

1.1.2. Confirmatory Factor Analysis: Using CFA, adequacy of the 14-item two-factor model was evaluated to ensure the adequacy of competing models: (1) the 22-item one-factor model (13) and (2) the 13-item one-factor model (20). The sample’s data appeared to be moderately asymmetric, as the largest skew and kurtosis were –1.33 and 1.74, respectively. Significant multivariate skewness and kurtosis indicated violation of multivariate normality assumption. The 14-item two-factor model showed acceptable fit (RMSEA = 0.059, 90% CI = 0.045 - 0.073; CFI = 0.96; TLI = 0.96; WRMR = 0.89), except for the significant chi-square test (χ276 = 148.5, p < 0.001). In contrast, the 2 competing models yielded unacceptable fits to the data (see Table 2).

1.1.3. Internal Consistency and Sex Differences: Table 3 separately summarizes the Cronbach’s alpha coefficients and sex differences for the exploratory and validation datasets. All Cronbach’s alpha values were acceptable, with the lowest being 0.65 corresponding to the social/emotional factor of the exploratory dataset. This was lower than the acceptable level perhaps due to having only 6 items. Item-total correlations were all above 0.25, except for item 12 in social/emotional subscale, which was 0.22 for the exploratory dataset and .19 for the validation dataset. No significant sex differences were reported although women scored slightly higher than men in all comparisons (Table 3). 

*1.2* *.* *SQ-Short–F*

1.2.1. Exploratory Factor Analysis: Univariate distribution of the observed variables showed a moderate asymmetry because the largest skew and kurtosis were both –1.10. Both multivariate skewness and kurtosis were statistically significant yielding violation of multivariate normality assumption.

Inspecting primary pattern coefficient matrix generated by EFA, revealed several items with cross-loadings, leading us to choose direct quartimin over promax rotation (27). According to eigenvalues and scree plot, either a one- or a four-factor model seemed adequate. Thus, models with number of factors ranging from 1 to 5 were evaluated to identify the best fit model. RMSEA for models with all 25 items of the SQ-Short–F loaded onto one factor was 0.07, two factors 0.07, three factors 0.06, four factors 0.05, and five factors 0.04, with no negative residual variances. Hence, a four- or five-factor solution was suggested to adequately fit the data. 

Once pattern coefficients of items of the four-factor model were reviewed, 3 items (7, 18, and 23) constrained the model interpretability perhaps due to misleading translations. For instance, item 23 (When I’m in a plane, I do not think about the aerodynamics) appears to point out the structural aspects of a plane in the original language. However, the Farsi translation of this item put emphasis on the flight and navigation rather than the aircraft structure and the motion of air. Similar possible misconceptions were noticed for the other 2 items. Such semantic differences could create difficulties for the structural modeling. Hence, to stick to original meanings of items and to increase the replicability of the results, these 3 items were removed from the analysis. EFA with WLSMV estimator and direct quartimin rotation was then repeated on the remaining 22 SQ-Short–F items loaded onto four factors. Factor 1 consisted of items 2, 4, 6, and 14 that could be regarded as pattern/strategy, except for item 2 (If there was a problem with the electrical wiring in my home, I’d be able to fix it myself.), which is a DIY item. It was also significantly loaded on factor 3 and was thus removed from subsequent analysis. Factor 2 consisted of items 3, 8, 10, 12, 13, 15, 17, 19, and 25 that could be conceptualized as “technicity”, except for items 10, 12, and 15. Item 10, 12, and 15 seemed to be related to structure, science, and DIY, respectively. Plus, they showed cross-loadings, which led us to eliminate them from the analysis. Factor 3 involved items 9, 11, and 22, which could be best described as topography. Factor 4 involved items 10, 21, and 24 that could be regarded as natural systems. The remaining 3 items (1, 5 and 16) were removed due to insignificant cut-off pattern coefficients. There were 3 more items (8, 9 and 11) that exhibited significant cross-loadings, of which; items 8 and 11 had pattern coefficients low enough to ignore these cross-loadings. Pattern coefficients of item 9 loaded onto factor 2 and 3 were 0.39 and 0.55, respectively. Because with items 11 and 22, a meaningful factor (factor 3) was formed and to stick to the three-indicator-per-factor recommendation (21), this item was retained. RMSEA for the remaining 15 SQ-short–F items loaded onto four factors dropped from 0.05 to 0.04, with no negative residual variances, showing significant improvement in the model fit (Table 4). There were significant (at p < 0.05) intercorrelations between factors 1 and 2 (r = 0.27), factors 2 and 3 (r = 0.28), factors 2 and 4 (r = 0.32), and factors 3 and 4 (r = 0.30; see Table 3).

Regarding the five-factor model, we could not find a meaningful structure even after modifying the model by removing items with insignificant or low pattern coefficients or cross-loading. Therefore, this model was rejected and the 15-item four-factor model was suggested as the best fit model.

1.2.2. Confirmatory Factor Analysis: In this phase, adequacy of the 15-item four-factor model and 3 competing models were tested: (1) the 25-item one-factor model (13); (2) the 13-item one-factor model (20); and (3) the 18-item four-factor model (10). Because items 7, 18, and 23 had been removed in the exploratory mode due to misleading Farsi translations, adequacy of the competing models was tested with and without these 3 items. Therefore, it is not surprising if you find 2 sets of goodness-of-fit indices for each competing model in Table 5. The largest skewness and kurtosis values for the current sample were –1.17 and –0.97, respectively, indicating a moderately asymmetric distribution. Multivariate normality assumption was violated due to statistically significant multivariate skewness and kurtosis statistics. 

Regarding the 15-item four-factor model, the following fit statistics were reported: χ284 = 184.3, p < 0.001; RMSEA = 0.07, 90% CI = 0.05, 0.08; CFI = 0.90; TLI = 0.88; WRMR = 1.01. The modification indices suggested that the model will be further improved if items 3 (I rarely read articles or web pages about new technology.) and 17 (I find it difficult to understand information the bank sends me on different investment and saving systems.) were loaded onto both factors 1 and 2. Goodness-of-fit indices for this model were as follow: χ282 = 152.6, p < 0.001; RMSEA = 0.06, 90%CI = 0.04, 0.07; CFI = 0.93; TLI = 0.91; WRMR = 0.90, except for the chi-square statistic that was statistically significant. All other indices were in favor of a relatively acceptable model. There were significant (p < 0.05) intercorrelations between factors 1 and 2, 1 and 3, and 1 and 4 at r = 0.70, 0.58, and 0.34, respectively, and between factors 2 and 3, and 2 and 4 at r = 0.65 and 0.51, respectively, and between factors 3 and 4 at r = 0.34 (Table 5).

None of the competing models could provide an acceptable fit to the data (see Table 5). The 16-item four-factor model adopted from the 18-item four-factor model of Ling et al. (10), in which the 2 problematic items were excluded, was discarded due to the occurrence of Heywood case. 

1.2.3. Internal Consistency and Sex Differences: Cronbach’s alpha coefficient for the SQ-short-F subscale scores was below 0.7 and it was 0.73 for the total score across the exploratory and validation datasets (Table 3). Given the fact that factors 1, 3, and 4 each had 3 items and factor 2 had 6 items, we interpreted these alpha values as acceptable. Across the 2 subsamples, item-total correlations were above 0.25, except for item 7 from the validation dataset (item-total correlation = 0.18). There were significant sex differences in the total SQ-short-F and subscale scores, except for the natural systems subscale across both halves of the study sample (Table 3).

**2** **.** **Association between EQ-short-F and SQ-short-F, and Test-retest Reliability**

There were weak to moderate positive correlations between the total scores of the 14-item EQ-short-F and 15-item SQ-short-F on the exploratory dataset (r = 0.24, p < 0.001; men, r = 0.27, p < .001; women, r = 0.25, p < 0.001) and validation dataset (r = 0.37, p < 0.001; men, r = 0.42, p < 0.001; women, r = 0.37, p < 0.001). Acceptable test-retest reliability was indicated by ICC values of 0.72 for the EQ-short–F (95%CI, 0.45 - 0.86) and .91 for the SQ-short–F (95%CI, 0.82 - 0.95).

**Table 1:** Final Pattern Coefficients, Eigenvalues, and Interfactor Correlation for the Promax-Rotated 14-Item Two-Factor Solution of the EQ–Short-F

	**Factor**	
	**1**	**2**
Eigenvalue	4.28	1.89
Promax-rotated pattern coefficient		
3. I find it hard to know what to do in a social situation.	0.51	0.14
4. I often find it difficult to judge if something is rude or polite.	0.53	0.07
6. I can pick up quickly if someone says one thing but means another.	–0.03	0.60
7. It is hard for me to see why some things upset people so much.	0.46	0.00
9. I am good at predicting how someone will feel.	0.17	0.56
10. I am quick to spot when someone in a group is feeling awkward or uncomfortable.	0.03	0.60
11. I can’t always see why someone should have felt offended by a remark.	0.48	–0.12
12. I don’t tend to find social situations confusing.	0.34	–0.01
16. I can sense if I am intruding, even if the other person doesn’t tell me.	–0.00	0.53
17. Other people often say that I am insensitive, though I don’t always see why.	0.46	–0.12
18. I can tune into how someone else feels rapidly and intuitively.	0.04	0.69
19. I can easily work out what another person might want to talk about.	–0.02	0.75
20. I can tell if someone is masking their true emotion.	0.01	0.73
21. I am good at predicting what someone will do.	–0.05	0.78
Inter-factor correlation		
Factor 1		
Factor 2	0.28*	

Note.—Entries in bold are statistically significant pattern coefficients. Factor 1: social/emotional; Factor 2: cognitive empathy.

* Statistically significant at p < 0.05.

**Table 2:** Summary of CFA Outputs for the 14-Item Two-Factor EQ-Short-F and the Competing Models

**Model**	**Item**	**Goodness-of-Fit Indices**								
		**χ** ^2^	**df**	**P**	**TLI**	**CFI**	**RMSEA**	**90% CI**	**p (close fit)**	**WRMR**
One-factor†	22	522.4	209	<0.001	0.89	0.88	0.07	0.07, 0.08	<0.001	1.26
One-factor‡	13	188.5	65	<0.001	0.94	0.95	0.08	0.07, 0.10	<0.001	1.06
Two-factor	14	148.5	76	<0.001	0.96	0.96	0.06	0.05, 0.07	0.14	0.89

Note. — χ2: Chi-square test; df: Degrees of freedom; TLI: Tucker-Lewis index; CFI: Comparative fit index; RMSEA: Root mean square error of approximation; WRMR: Weighted root mean square residual.

† This model was suggested by Wakabayashi et al. (13).

‡ This model was suggested by Samson et al. (20).

**Table 3:** Sex Difference and Cronbach’s α for the Total and Subscale Scores of the 14-Item Two-Factor EQ-Short-F and 15-Item Four-Factor SQ-Short-F and for the Exploratory and Validation Datasets Separately

	**Exploratory Dataset (n = 271)**					**Validation Dataset (n = 271)**				
	**Male,** **Mean ** **(SD)**	**Female,** **Mean ** **(SD)**	**t**	**d**	**α**	**Male,** **Mean ** **(SD)**	**Female,** **Mean ** **(SD)**	**t**	**d**	**α**
**14-item two-factor EQ-short-F score**										
Total	13.6 (5.38)	14.0 (4.54)	-0.8	0.08	0.76	13.5 (5.47)	13.9 (5.32)	0.6	0.07	0.82
Factor 1 (Social/emotional)	5.2 (2.76)	5.6 (2.51)	-1.1	0.14	0.65	5.1 (2.75)	5.4 (2.76)	0.6	0.10	0.72
Factor 2 (Cognitive empathy)	8.4 (3.97)	8.5 (3.27)	-0.3	0.04	0.82	8.3 (3.79)	8.5 (3.66)	0.4	0.05	0.85
**15-item four-factor SQ-short-F score**										
Total	15.7 (5.66)	13.17 (5.30)	3.8**	0.47	0.75	16.3 (5.07)	13.4 (5.06)	4.5**	0.57	0.73
Factor 1 (Pattern/strategy)	2.7 (1.74)	2.4 (1.51)	2.1*	0.25	0.50	2.9 (1.55)	2.3 (1.52)	2.8*	0.36	0.50
Factor 2 (Technicity)	6.9 (2.71)	5.6 (2.88)	3.6**	0.55	0.68	7.4 (2.42)	5.6 (2.66)	5.3**	0.68	0.65
Factor 3 (Topography)	3.4 (1.80)	2.5 (1.69)	4.2**	0.52	0.63	3.4 (1.75)	2.6 (1.69)	3.9**	0.49	0.64
Factor 4 (Natural systems)	2.6 (1.82)	2.6 (1.62)	-0.1	0.01	0.60	2.6 (1.65)	2.9 (1.51)	-1.3	0.16	0.56

Note. t, t statistic calculated using independent samples t test; d, Cohen’s d; α, Cronbach’s alpha coefficient; EQ-short-F developed by Wakabayashi et al. (13): factor 1 consisted of items 3, 4, 7, 11, 12, and 17; factor 2 consisted of items 6, 9, 10, 16, 18, 19, 20, and 21. EQ-short-F developed by Wakabayashi et al. (13): factor 1 consisted of items 4, 6, and 14; factor 2 consisted of items 3, 8, 13, 17, 19, and 25; factor 3 consisted of items 9, 11, and 22; and factor 4 consisted of items 20, 21, and 24.

* P < 0.05

** P < 0.001

**Table 4:** Final Pattern Coefficients, Eigenvalues, and Interfactor Correlations for the Quartimin-Rotated 15-Item Two-Factor Solution of the SQ–Short-F

	**Factor**			
	**1**	**2**	**3**	**4**
Eigenvalue	3.92	1.69	1.39	1.23
Quartimin-rotated pattern coefficients				
3. I rarely read articles or web pages about new technology.	0.32	0.42	–0.13	–0.04
4. I do not enjoy games that involve a high degree of strategy.	0.56	0.03	–0.02	–0.06
6. In math, I am intrigued by the rules and patterns governing numbers.	0.59	0.02	0.07	0.01
8. If I were buying a computer, I would want to know exact details about its hard disc drive capacity and processor speed.	0.02	0.71	–0.09	0.05
9. I find it difficult to read and understand maps.	0.01	0.29	0.54	–0.15
11. I find it difficult to learn my way around a new city.	–0.05	–0.05	0.77	0.03
13. If I were buying a stereo, I would want to know about its precise technical features.	0.14	0.51	0.08	0.18
14. I find it easy to grasp exactly how odds work in betting.	0.39	0.11	0.04	0.04
17. I find it difficult to understand information the bank sends me on different investment and saving systems.	0.08	0.29	0.09	0.03
19. If I were buying a camera, I would not look carefully into the quality of the lens.	0.07	0.47	0.15	0.02
20. When I hear the weather forecast, I am not very interested in the meteorological patterns.	–0.09	0.35	0.16	0.23
21. When I look at a mountain, I think about how precisely it was formed.	–0.13	0.10	0.02	0.80
22. I can easily visualize how the motorways in my region link up.	0.14	–0.04	0.63	0.11
24. I am interested in knowing the path a river takes from its source to the sea.	0.20	–0.07	0.02	0.68
25. I am not interested in understanding how wireless communication works.	–0.04	0.73	0.04	–0.10
Inter-factor correlation, Factor				
1				
2	0.27*			
3	0.12	0.28*		
4	0.07	0.32*	0.30*	

Note.—Entries in bold are statistically significant pattern coefficients. Factor 1: pattern/strategy; factor 2: technicity; factor 3: topography; factor 4: natural systems.

* Statistically significant at p < 0.05.

**Table 5:** CFA Result Summary for the 15-Item Four-Factor SQ–Short-F and the Competing Models

**Model**	**Item**	**Goodness-of-Fit Indices**								
		**χ** ^2^	**Df**	**p**	**TLI**	**CFI**	**RMSEA**	**90% CI**	**p-value (close ** **fit)**	**WRMR**
One-factor†	25	664.2	275	<0.001	0.78	0.80	0.07	0.07, 0.08	<0.001	1.40
	22	537.6	209	<0.001	0.80	0.82	0.08	0.07, 0.08	<0.001	1.38
One factor‡	13	171.0	65	<0.001	0.82	0.85	0.07	0.06, 0.09	0.001	1.15
	12	139.7	54	<0.001	0.85	0.88	0.08	0.06, 0.09	0.003	1.09
Four-factor§	18	285.0	129	<0.001	0.86	0.88	0.07	0.06, 0.08	0.005	1.13
Four-factor	15	152.6	82	<0.001	0.91	0.93	0.06	0.04, 0.07	0.21	0.90

Note. — χ2: Chi-squared test; df: Degrees of freedom; TLI: Tucker-Lewis index; CFI: Comparative fit index; RMSEA: Root mean square error of approximation; WRMR: Weighted root mean square residual.

† This model was suggested by Wakabayashi et al. (13). Three ambiguously translated items (7, 18, and 23) were removed from the 25-item SQ-short, and the remaining 22 items were fitted against a one-factor model (for more details see text).

‡ This model was suggested by Samson et al. (20). Item 7, which was ambiguously translated, was eliminated from the 13-item SQ, and the remaining 12 items were fitted onto a unifactorial structure (for more details see text).

§ This model was suggested by Ling et al. (10).

## Discussion

Applying a cross-validation design, the present study investigated the factor structure and psychometric properties of the Farsi versions of the EQ–Short and the SQ–Short in a nonclinical sample of Iranian university students. EFA on the exploratory dataset resulted in a 14-item two-factor model for the EQ-Short–F and a 15-item four-factor model for the SQ-short-F. Using the validation dataset, CFA confirmed the factorial validity of the models identified by EFA. Regarding the EQ-short-F, factor 1 (social/emotional) involved 3 emotional reactivity items (7, 11, 17) and 3 social skills items (3, 4, 12) and factor 2 (cognitive empathy) consisted of 8 items (6, 9, 10, 16, 18, 19, 20, 21). SQ-short-F factors were as follow: factor 1: pattern/strategy (items 4, 6, 14), factor 2: technicity (items 3, 8, 13, 17, 19 25), factor 3: topography (items 9, 11, 22), and factor 4: natural systems (items 20, 21, 24). Items categorized into these factors are comparable to those of previous studies (8-10, 15). Additionally, acceptable internal consistencies across the study subsamples and adequate test-retest reliability were demonstrated for the suggested models.

Regarding the 15-item four-factor SQ-short-F across the exploratory and validation datasets, significant sex differences were found for the total and pattern/strategy, technicity, and topography subscales, with men scoring higher than women. These differences showed reasonable observed power ranging from 0.50 to 0.75. Unexpectedly, total and subscale scores of the 14-item two-factor EQ-short-F as well as natural systems subscale of the SQ-short-F failed to show significant gender differences. These insignificant comparisons demonstrated low observed powers (ranging from 0.05 to 0.16, Cohen’s d) suggesting the lack of evidence in support of the null hypothesis. However, such an interpretation seems to be flawed, as observed power is a function of observed p-value (28). Instead, we proposed that these unexpected findings can be explained by constraints of our study sample: (1) a whole nonclinical sample, (2) volunteer participation, and (3) male to female ratio of 1:2, which although reflects the sex ratio of the population of Iranian university students, restricts the range of expected values.

The association between empathizing and systemizing has been open to debate, so as the correlation between the scores of the EQ and SQ (coefficients, -0.28 to 0.22) (16, 17, and 29). This study yielded a weak to moderate positive correlation between the scores of the EQ-short–F and the SQ-short–F over the halves of the study sample. Furthermore, we found that the correlation was stronger for men compared to women, which might suggest that this association is sex-dependent (30). 

We proposed that factor structures identified for the EQ and SQ by previous investigations were limited in replicability. For instance, the 28-item three-factor version of the EQ developed by Lawrence, et al. (8) failed to be replicated by others (9, 15, and 31). As another example, unifactorial constructs for the SQ suggested by early studies (3, 8) were also rejected by some later research (10). The limited replicability of these constructs may come from inappropriate decisions made during the process of factor analysis. Our methodology was adopted to address such improper choices. CFA on the validation dataset could acceptably reproduce the models identified by EFA on the exploratory dataset, supporting the soundness of the proposed methodology.

## Limitation

The validation course of action is an ongoing procedure and this study is limited by lacking concurrent validity of EQ or SQ with other related scales. 

## Conclusion

In conclusion, the present study provided preliminary evidence to support the adequate factorial validity and reliability of the 14-item two-factor EQ-short-F and 15-item four-factor SQ-short-F questionnaires. Despite the use of different statistical approaches in the decisions made in the process of factor analysis, relatively comparable results were obtained when comparing the results of the present study with those of previous reports on the factor structure of the EQ and SQ. The present study adds to previous investigations suggesting multifactorial structures for the EQ and SQ.
